# The five pillars of computational reproducibility: bioinformatics and beyond

**DOI:** 10.1093/bib/bbad375

**Published:** 2023-10-21

**Authors:** Mark Ziemann, Pierre Poulain, Anusuiya Bora

**Affiliations:** Deakin University, School of Life and Environmental Sciences, Geelong, Australia; Burnet Institute, Melbourne, Australia; Université Paris Cité, CNRS, Institut Jacques Monod, Paris, France; Deakin University, School of Life and Environmental Sciences, Geelong, Australia

**Keywords:** reproducibility, computational research, bioinformatics, research best practices

## Abstract

Computational reproducibility is a simple premise in theory, but is difficult to achieve in practice. Building upon past efforts and proposals to maximize reproducibility and rigor in bioinformatics, we present a framework called the five pillars of reproducible computational research. These include (1) literate programming, (2) code version control and sharing, (3) compute environment control, (4) persistent data sharing and (5) documentation. These practices will ensure that computational research work can be reproduced quickly and easily, long into the future. This guide is designed for bioinformatics data analysts and bioinformaticians in training, but should be relevant to other domains of study.

## INTRODUCTION

In research, computational reproducibility is the ability to use the materials from a past study (such as data, code and documentation) to regenerate the outputs including figures and tables to confirm the study’s findings [1]. Reproducibility is only the first step toward overall reliability; for example, a study may be reproducible, but suffer from analytical problems that invalidate the results. If a study is reproducible, then at least these analytical issues can be more easily identified and rectified. Therefore, reproducibility is a necessary stepping-stone in the framework of cumulative science. Reproducibility should enhance the overall reliability of computational research including replicability and robustness. Replicability being defined as the ability to repeat the entire study and come up with similar overall results. Robustness refers to the ability for the findings to be generalizable to other situations [2].

Much has been written on how irreproducibility has reached crisis levels in psychology and life sciences [3, 4]. Indeed, in bioinformatics, the situation is grim with a 2009 systematic evaluation showing only 2 of 18 articles could be reproduced (11%) [5], bringing into question the reliability of those studies. Workshops conducted by National Institutes of Health intramural researchers in 2018/2019 sought to reproduce five bioinformatics studies and could not reproduce any, citing missing data, software and documentation [6]. A recent systematic analysis of Jupyter notebooks in biomedical articles had similar observations, with only 245/4169 notebooks (5.9%) giving similar results compared to the original, with irreproducibility blamed on missing data, broken dependencies and buggy code [7]. A similar survey of R scripts in the Harvard Dataverse repository found slightly better results, with 26% of scripts completing without errors [8].

The ramifications of irreproducible and unreliable research includes misleading the community, wasting research funds, slowing scientific progress, eroding public confidence in science and tarnishing the reputation of associated institutions and colleagues. In clinical research, irreproducible bioinformatics has the potential to place patient safety at risk.

For example, in 2006 an article entitled ‘Genomic signatures to guide the use of chemotherapeutics’ generated a great deal of interest as it was an early application of high-throughput transcriptomics in the prediction of individual patient responses to different chemotherapies [9]. After observing some unusual features of the patient group, Baggerly and Coombes [10] attempted reproduction of some of the key findings. Without the help of scripted workflows to guide the re-analysis, the team used forensic bioinformatic techniques to piece together how the dataset was originally analyzed. Their investigations found a litany of issues. Firstly, the labeling of patients in the test set as ‘responders’ or ‘non-responders’ had been reversed in the original analysis. Secondly, some of the patients were included more than once (some up to four times) in the analysis, likely to cause major distortions in results. Confusingly, some of the reused data has inconsistent grouping, i.e. some of the samples are labeled both sensitive and resistant. Additional errors include two cases where results (charts) were ascribed to the wrong drug. Baggerly and Coombes highlight that such mistakes can inadvertently occur when conducting unscripted data analysis such as using spreadsheets, and these problems can be obscured by a lack of documentation. The article underwent two corrigenda, but was ultimately retracted in 2011, as the authors were not able to reproduce the findings themselves due to ‘corruption of several validation data sets’ [11]. As the array findings were the basis for clinical trials where patients were allocated to treatments, the flawed data analysis may have harmed patients given the wrong drug in the period 2007–10. In 2010, Duke University terminated the trials and suspended the lead author, Dr Anil Potti, who later resigned. Duke was served eight lawsuits by families of affected patients seeking compensation for exposure to harmful and unnecessary chemotherapy, which were settled out of court [12]. This worst-case scenario emphasizes that computational reproducibility is crucial for translating bioinformatics research into real-world outcomes.

## RECOMMENDATIONS

**Figure 1:**
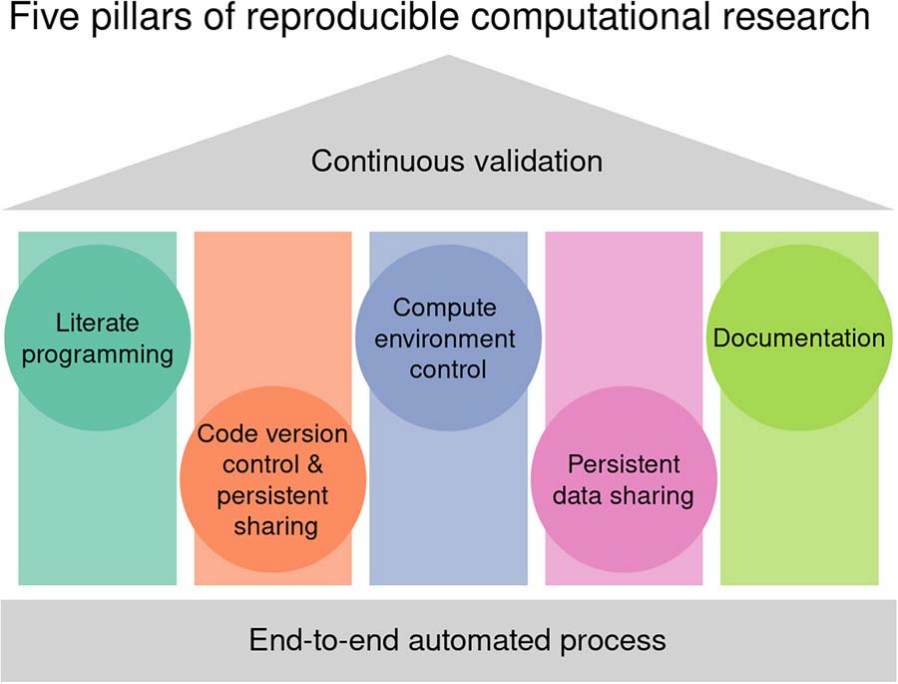
The five pillars of computational reproducibility.

A number of guides recommending enhanced computational reproducibility practices have been developed [13–28]. Inspired by these principles, we present the five pillars of reproducible computational research (Figure 1). Here, the emphasis is on practical reproducibility with an increased focus on programming practices, transparent reporting and the role of computational environments. The intended audience is bioinformatics data analysts and bioinformaticians in training; however, the principles described here could equally apply to other domains of study. There are clear parallels with the established three pillars of the open science framework, (open data, code and papers) [29].

### End-to-end automated process

To ensure reproducibility of bioinformatics workflows, they need to be formalized in code wherever possible, from inspecting the raw data to generating the outputs that form the conclusions of the study. Automated processes remove the need for manual steps, which are time-consuming and prone to errors. Without an end-to-end automated process, most of the other best practices described here are not possible. Scripted workflows, although not always free of errors, enable better auditing and easier reproduction, which would be difficult for graphical tools like spreadsheets or web tools. Indeed, spreadsheets are prone to data entry, manipulation and formula errors [30], leading to problems like inadvertent conversion of gene names to dates in databases and Supplementary Excel files available online at http://bib.oxfordjournals.org/ [31–33]. Spreadsheet errors could be widespread, given that it is used as an analysis tool by ~69% of researchers according to a survey undertaken in 2015–16 of 20 000 university academics [34].

While web tools are valuable for data exploration, there are worries that they undermine reproducibility for the sake of convenience [35]. Transferring data between compute platforms is also discouraged. For example, having workflows that involve combinations of web-based and scripted tools require data transfer steps that are inefficient and error-prone. On the other hand, some web-based tools excel at reproducibility. The web-based analysis platforms Galaxy and GenePattern enable sophisticated point-and-click bioinformatics analysis in the browser, and those workflows can also be shared in a reproducible way [36, 37]. Some web tools facilitate reproducibility by providing code (e.g. Degust [38]) or by allowing apps to be executed locally (e.g. ShinyGO [39]).

To facilitate an end-to-end automated process, code and data need to be ‘linked’, which means the code is aware of the location of the data and how to get it automatically [14]. The code should be able to fetch the data from a publicly accessible location and conduct the downstream processing and analysis without having to acquire the data some other way.

A caveat for end-to-end automation is that sometimes, manual data cleaning is unavoidable. In those cases, retaining raw and cleaned data along with a cleaning protocol is recommended. Then, the computational workflow can begin with the cleaned dataset.

Another issue is that perfect reproducibility isn’t possible in cases where some element of randomness is required by the underlying algorithms. Such algorithms are extensively used in molecular simulation, machine learning, permutation-based statistical tests and certain data projection methods, namely, t-distributed stochastic neighbor embedding (t-SNE) [40] and uniform manifold approximation and projection (UMAP) [41], which are popular for visualizing high-dimensional omics data [42]. To make such workflows deterministic, the pseudo-random number generator can be initialized with a fixed value (sometimes called ‘setting the seed’) [15]. However, this needs to be done with care to ensure the results do not misrepresent the bulk of iterations [43].

A guiding principle of the five pillars approach is that the publishing researchers should strive to make the reproduction process easier and faster for those who wish to. A ‘master script’ that coordinates the execution of individual parts of an analysis is an excellent way to reduce the number of commands required for reproduction. This can be coded in standard open-source languages like R/R Markdown, Python/Jupyter notebook or the Unix Shell. These approaches work equally well on personal computers, cloud servers and high-performance clusters. Using free and open-source software ensures that the research is accessible to the greatest audience [44], as opposed to proprietary software like SPSS, STATA and MatLab, which are cost inhibitory. Free and open source software also enjoy relatively larger libraries of add-on packages contributed by the scientific community. Projects involving computationally intensive tasks would benefit from a build/workflow automation solution. Guides for selecting and using such systems have been already reviewed by others [45, 46]. But it is worth mentioning some of the most used solutions in bioinformatics, which include snakemake [47, 48], targets [49], CWL [50], WDL [51] and nextflow [52]. The advantage of such tools is that if the analysis were to terminate due to an issue midway, for example, a hardware problem at step 8 of a 15-step workflow, the analysis of steps 1–7 wouldn’t need to be repeated. After fixing the issue, re-running the analysis would pick up at step 8 again, which saves labor and compute time.

### Literate programming

Literate programming combines ‘chunks’ of analytical code with human-readable text [53]. After compilation, the resulting output document contains the code together with computational results such as figures and tables along with contextualizing explanations and narratives.

**Figure 2:**
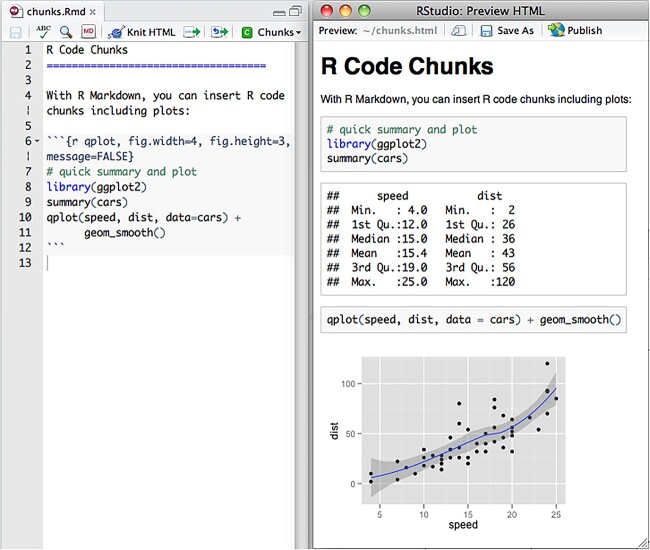
An example R Markdown script (left) and output document (right) [55].

The Sweave project, conceived in 2001, was designed to give LaTeX documents embedded R code chunks and was envisaged as a way to generate R documentation materials and generate statistical analysis reports [54]. Around 2015, R Markdown emerged as a more popular alternative, as formatting content is simpler with Markdown as compared to LaTeX, saving time. An example R Markdown script and output report is given in Figure 2.

Around the same time, Project Jupyter was developed to provide a ‘notebook’-type interface, incorporating R, Python, Julia and other computer language chunks into documents [56–58]. A noteworthy attempt to use literate programming in Jupyter notebooks to aim for reproducibility has been the analysis of RNA-seq samples from patients infected by the Zika virus [59]. In this work, authors provided alongside the traditional research paper a Jupyter notebook that performed and documented the entire analysis procedure. The MyST (short for Markedly Structured Text notebook) is built on top of Jupyter notebooks or Markdown documents and has some notable advantages around flexibility of content that make it an attractive choice for executable article authoring [27].

In 2022, the company behind RStudio (Posit) released Quarto, the conceptual successor of R Markdown, but with enhanced support for other computer languages like Python, Julia, and Observable JavaScript [60].

Whether R Markdown, Jupyter or other system, literate programming offers some substantial benefits over alternative approaches:

(i) The provenance of any result can be demonstrated. The resulting document produced contains the code executed, together with the results (e.g. a chart). This is in contrast to a data analysis report assembled in a word processor, which, due to the many copy-paste operations required, might contain errors or version mismatches.
(ii) It saves time. For the case where a report needs to be run routinely, this would involve significant copy-paste to be assembled ‘manually’ with a word processor. Using a literate script would mean only minimal changes are required between iterations [61].
(iii) It accommodates extensive documentation. This allows the analyst to include rich descriptions of scientific works. For example, a report may contain background, methods, results, discussion and references. Not only text but also various objects can be included like links to other resources, tables, images, videos, etc. This means it is possible to author an entire journal article using literate programming.
(iv) Outputs are arranged. When using a regular script, an analyst might make dozens of charts which are written to the disk with names like ‘chart1.png’ or ‘model8.svg’. When the number of outputs is large, it becomes unwieldy and hard to place which plot corresponds to which part of the script. By embedding the outputs such as charts and tables in a document in the sequence that they were generated, it helps the reader understand the logical steps taken in an analysis.
(v) Amenable to version control. Version control is a useful best practice in software development (discussed below).
(vi) Output reports are free from code errors. The output document is only rendered when the entire script has been compiled without errors. In contrast, a regular script might generate some outputs and then encounter an error, so we are not sure whether the whole script is free of errors and completed successfully. Therefore, it is good practice to routinely execute these scripts during the development process and not rely too heavily on the interactive execution of individual chunks or lines.
(vii) Flexible output formats. These include PDF, DOC/DOCX and HTML, the latter having some notable benefits including better usability for mobile devices like phones and tablets and the ability to support richer content such as interactive charts and dynamic tables (searchable, filterable, sortable). Outputs include not only HTML documents but also slideshows and even e-books. Quarto and R Markdown have the ability to automatically generate a bibliography in many different journal styles [62]. Further, dozens of journal-style Markdown document templates are freely available and integrate with the MyST notebook system or the rticles R package [63].

These features make literate programming a useful tool for science communication in a range of situations. Whether this is sharing a data analysis report, giving a presentation at a meeting, writing a research article or self-publishing an e-book, literate programming provides the ability to construct transparent data narratives with clear provenance in a conveniently shareable form.

Literate programming also works neatly with the concept of the ‘executable paper’, the idea that the data analysis underlying an entire study can be reproduced with one or a few commands [64–66]. A typical genomics/bioinformatics study could involve one large literate script, or be broken down into smaller scripts, where each one contains the code for generating a part of the article. A multi-script approach would benefit from a master script that executes each component. This further makes the job of reproducibility easier.

While it is common to run literate programs on a PC with graphical interfaces such as RStudio, they can also be executed in ‘headless’ mode on high-performance computing clusters or cloud servers using command-line interfaces to take advantage of greater computational resources. The headless mode is also important for integrating literate scripts into larger workflows and for automated testing.

### Code version control and persistent sharing

**Figure 3:**
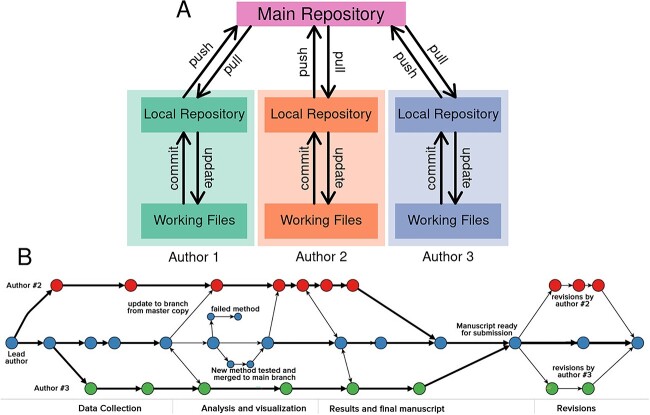
Distributed version control. (**A**) Each author has a copy of the repository where their contributions are committed before being pushed to the main central repository. Adapted from [70]. (**B**) An example hypothetical git workflow history for a research project involving a team of three authors. Circles represent code commits. Path divergences create separate branches for independent development. Horizontal paths indicate code changes for a particular branch. Path convergences indicate where branch specific differences are incorporated into the main branch. From [71].

In bioinformatics, sharing code is becoming standard practice for reproducibility and transparency and is a requirement for many specialized journals [67]. Code sharing appears to improve the rate of article citations [68]. One of the most popular ways to share research code is through online software repositories with integrated version control [69]. A version control system (sometimes called ‘source control’) is a type of program that ‘tracks changes’ made to sets of files, typically other computer program source code, scripts and documentation. A repository is simply a set of files under version control that represents a project or sub-project. Version control is used extensively by software developers and is considered one of the key best practices in software engineering. Distributed Version Control Systems (DVCS) involve a central web-accessible server hosting a repository, and each team member possesses a mirror copy on their local system (Figure 3). Having a central, publicly available node assists in disseminating changes within teams and releasing code to consumers. There are many such DVCSs (Subversion, Git, mercurial, etc.), but git has emerged as the most popular solution due to its many powerful features, speed/efficiency and large community and ecosystem.

Although DVCSs come from the world of software engineering, data analysts can significantly benefit from their use in the life sciences [71–73]. Below, we outline some of these advantages.

(i) Keeps a complete history of all code changes over time. We always know which version of the script is the most up to date. We can inspect and execute the code and reproduce the analysis at any particular point in the past. Commit messages are retained, which gives a record of the motivations, purpose and person behind each modification.
(ii) Helps collaboration and project management. Using a centralized code hosting platform such as ‘GitHub’ or ‘BitBucket’ can help manage contributions from team members who may be working asynchronously in different time zones and on different servers. It also makes the user’s work searchable, which is convenient when code snippets from a project need to be reused for a new project.
(iii) Helps resolve issues. These centralized platforms also enable discussions of code issues such as bugs and feature requests. In bioinformatics teams, issues can be used to track the progress of a project through its milestones and allow team members to raise potential issues with the code. This also allows the scientific community to raise an issue with the software maintainers. For example, this feature is used extensively during the peer review process for the *Journal of Open-Source Software* [74].
(iv) Supports documentation best practices. Books, tutorials, protocols and other educational resources are well served using DVCS (see below).
(v) Makes works easily shareable. Work is easier to reproduce because the source folder contains a complete workflow with the code linked to data and metadata, so it doesn’t need any modifications before reproduction. It is relatively easy to add a software license with a DVCS like GitHub, so consumers can understand the conditions of using it.
(vi) Protects against code loss. Computers sometimes fail and we sometimes delete or overwrite important files. If code is lost and unrecoverable, it can be a significant cost to replace. Although DVCSs are not a back-up solution, they do add a layer of protection to the code. Preserving a copy of the repository on each team members’ computer in addition to the central repository means the code can be easily recovered if any one of those computers fails.

Although DVCSs assist with code sharing, they are not considered a long-term archiving solution, as recalled by the complete shutdown of the Google Code platform in 2015 and the end of Mercurial support by the Bitbucket platform in 2020. The code needs to be deposited to a long-term repository. Zenodo and Figshare are often used to store the content of a repository but do not retain the history of the development of the source code. Software Heritage [75, 76] is the universal software archive whose mission is to collect, preserve and share source code. Software Heritage provides an intrinsic persistent identifier called SWHID that allows to cite the archived source code in the respective journal article/preprint [77]. Archiving source code in Software Heritage can be performed manually or automatically using a webhook within continuous integration.

git is typically used at the command line; however, it is also incorporated into integrated development environments commonly used in bioinformatics including RStudio, JupyterLab [78, 79] and VS Code [80]. There are also several git clients with graphical interfaces that better allow inspection of code changes (e.g. [81, 82]).

### Compute environment control

Most popular software undergoes regular updates to patch bugs and add new features. Bioinformatics software is no different, and it is well known that such changes have the potential to affect results [83]. This is why it is best practice to report the exact version of all programs used in an analysis (and packages therein) and even make archival copies for future reference [15].

In R, such reporting is possible using the sessionInfo() command, while for Python, this is possible using the session_info or watermark packages. Using literate programming and sharing output documents ensure that a record of this important information is made available.

Although regular software updates are overall a good thing, it poses a problem for future reproducibility. A researcher trying to reproduce a 10-year-old study in R v3.0 could have a headache, as they would need to roll back their R version, and possibly their operating system as well, as R and other languages require certain system dependencies for low-level routines [84].

To avoid this, a virtual machine (VM) could be used to run a system-in-a-system. This means that the ‘host’ machine can run another ‘guest’ operating system with the right R version, without needing to change the host R version. While this provides good reproducibility, setting up an environment with a 10-year-old operating system, R and packages would take a few hours to accomplish. Researchers could take a snapshot of their VM system and share it to help reproducibility and auditability [85, 86]; however, the size of these images is relatively large due to the fact it contains the OS, software stack and project data. Moreover, the performance of computation in the guest system is typically slower than when run directly on the host.

**Figure 4:**
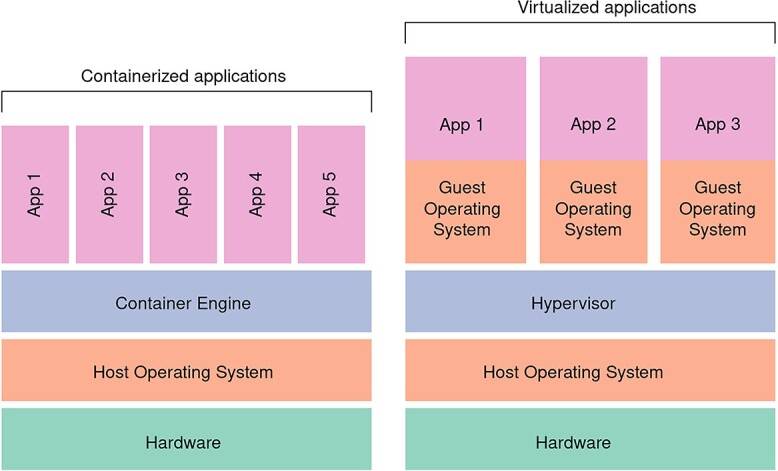
Architecture of containers (left) compared to VMs (right). Adapted from [88].

Containers are an attempt to resolve some of the downsides of VMs. Container images can be thought of as similar to VMs, but are more lightweight as they share parts of the host operating system [87]. In the example shown in Figure 4, the five containerized applications share the same operating system, while the three VM applications each involve their own operating system which incurs a significant performance overhead [88]. Therefore, running workflows in containers incurs only a small reduction in performance as compared to running directly on the host system [89]. Container images are highly portable because containers include everything needed to run the application, including the system tools and libraries, ensuring that the environment is consistent across different systems. For example, the most popular containerization system, Docker [90], makes it possible to run Windows and Linux/Unix containers on any computer with Docker installed, with the promise of reproducibility. While there are several alternatives such as Podman [91] or Apptainer/Singularity [92], Docker remains the most widely used containerization system (according to GitHub stars as of August 2023). Docker has a large community of users, extensive documentation and a vast collection of pre-built container images in the DockerHub registry. Docker can fetch images from DockerHub and run them on the host system with just a couple of commands, and typically within a few minutes. This accelerates the installation procedure dramatically, which is a known bottleneck for bioinformatic reproducibility [28, 93, 94]. In bioinformatics, containers are already extensively used. For example, BioContainers is a registry for sharing bioinformatics software containers [95]. Containers are equally useful to data analysts by encapsulating the environment in which analytical scripts are executed.

Another potential solution to this problem is to use a package/environment management system such as Conda or Guix. These allow users to create, manage and deploy software packages, dependencies and environments across different computing platforms [96]. Conda was initially developed to solve the problem of package dependency management in Python, but it now supports many other programming languages, including R, C/C++, Java and others. Conda allows researchers to create isolated environments with specific versions of packages, so users can have different versions of R or Python in different environments on the same host system. Conda environments have limitations around portability and consistency, as such environments are tied to specific operating systems and hardware architecture.

Guix is described as a ‘functional package manager’ and has the unique benefit of giving bit-for-bit build reproducibility and verifiability [84, 97]. This is a notable advantage, as Docker images are not verifiable nor guaranteed to build reproducibly in the future due to link decay. Moreover, Guix can build and output environments as Docker-compatible images, which solves the provenance problem of such environments [98]. These features are being used in the bioinformatics sphere to create highly reproducible pipelines that work equally well on personal as well as shared high-performance computers [97, 99]. Guix is among the best solutions for the ‘10-year challenge’ of code reproducibility [100]. Although Guix has some exciting functionality, there remains a relative lack of step-by-step guides and tutorials, illustrated by the complete absence of published Guix protocols in protocols.io, as compared to 13 and 12 related to ‘Conda’ and ‘Docker’, respectively (as of 30 May 2023). Lastly, it’s worth mentioning that the Guix project uses Software Heritage as a fallback system to provide a package source code if the download from its original location failed.

We are also watching with interest the early development of WebAssembly (Wasm)-based approaches for compute environment control. The ability to reproduce operating systems, programming languages and workflows in the browser opens up intriguing possibilities for more widespread reproduction and auditing without the need to install any software at all. Two notable examples of this include JupyterLite, a version of JupyterLab in early development that runs entirely in the browser [101], and WebR-enabled interactive code blocks in Quarto HTML documents [102].

It is not sufficient to simply use these tools; they need to be shared as part of the publication process. Conda environments are described by a file called environment.yml, which acts like a recipe for how to make the environment. Similarly, Guix environments rely on two files: channels.scm and manifest.scm. Docker images are built using a Dockerfile instruction set. These are small files that are easily shared in the project code repository. To enable rapid reproduction, making Docker images available is recommended. As these are often very large, they cannot be shared in the code repository; rather, they are commonly deposited to a Docker image registry such as DockerHub. One must keep in mind that availability of images is dependent on the commercial viability of Docker Inc, so it isn’t guaranteed to survive the 10-year challenge. To ensure long-term availability, it would be advised to deposit images to the BioContainers registry [95], as it is community driven and supported by consortia such as *Elixir* and *Global Alliance for Genomics and Health* that will underpin its longevity. Still, these are not considered permanent archival services, so it is advised to deposit the image used in an article to a suitable persistent long-term archive (discussed below).

### Persistent data sharing

Without data sharing, computational research is not reproducible nor auditable. Lack of data sharing is one of the key reasons why research is irreproducible [103]. Data sharing is also one of the key features of ‘open science’, which is characterized as science that is collaborative, transparent, accessible and inclusive [104, 105]. In addition to facilitating reproduction and auditing, sharing enables reuse in other contexts. Data reuse increases efficiency, as it prevents redundant research expenditure and facilitates new research ideas that were previously impossible [106]. In terms of research rigor, inspecting raw data can uncover inadvertent errors and research integrity problems [107]. In light of this, a research article without supporting data and code is much like a press release or advertisement where the claims made cannot be verified as true or not [27, 109].

Although it is common to see journal articles with ‘Data available upon reasonable request’, this is less than ideal. A systematic study of such data availability statements found that the data were successfully provided in just 6.8% of requests [109]. In the rare cases that data are shared in accordance with the data availability statement, it poses a burden in terms of labor to lodge and respond to such requests and, if the data are large, may incur an additional cost for hosting/transferring these large files.

The field of genomics has a long history of data sharing [110], which is supported by policy leadership by funding bodies [111, 112] and data sharing policies of journals (e.g. [113–115]). Best practices for research data sharing have been proposed [106], as have recommendations around sharing human genetic/genomic data [116]. While deidentification of data has long been used to safeguard participant privacy, this may not be suitable for genomics data due to the availability of existing public genetic data sets that heighten reidentification risks [117]. For example, the surnames of some male participants can be inferred based on the Y chromosome variants seen in deidentified genomic data together with public genealogy web sites [118]. To foster the responsible reuse of sensitive genomic and health data, the Global Alliance for Genomics and Health (GA4GH) initiative has proposed strategies, technical standards and policy frameworks designed to protect personal data in a way that preserves reproducibility [119, 120].

In order to maximize the value of shared data for reuse and reproducibility, it needs to be findable, accessible, interoperable and reusable (FAIR) for people and for computers [106]. In the wake of the Human Genome Project, a number of repositories for specific biological data types were established including Gene Expression Omnibus (GEO), Sequence Read Archive (SRA), European Nucleotide Archive (ENA) and Proteomics Identifications Database (PRIDE) [121–123]. Although these repositorie suffer issues around interoperability and reusability, they do support data reuse through findability and accessibility [124, 125]. The re3data.org registry of data repositories may be useful to find repositories that accepts data from other domains of study like ecology, physiology, molecular simulation, social sciences and computing [126]. If no specialized repository exists, then a general-purpose repository such as Dryad, FigShare or Zenodo should be considered. Researchers should be aware, however, that these repositories are often not moderated and that it is their responsibility to provide sufficient metadata. To this aim, general recommendations have been proposed to enhance data reuse in ecology, but which can also be applied to other disciplines [127].

Key recommendations include the following:

(i) Deposit data to a specialized repository if possible, otherwise, a general-purpose repository.
(ii) Avoid commodity cloud storage as these are impermanent and susceptible to link decay [128].
(iii) Avoid large Supplementary Data files available online at http://bib.oxfordjournals.org/ accompanying journal articles as these are less findable and accessible [129].
(iv) Preferably archive and share raw data and use existing standards for the discipline.
(v) Use file formats that are machine-readable and compatible with many different types of software. Some examples include comma- and tab-separated values (CSV/TSV) formats, eXtensible Markup Language (XML), JavaScript Object Notation (JSON), Hierarchical Data Format version 5 (HDF5) and Apache Parquet.
(vi) Provide detailed metadata, e.g. sample descriptions that match the article; describe the columns in tabular data (i.e. data dictionary).

Once the quality of the data set has been established, researchers may consider depositing it to a data repository early, before publicization, as it has some advantages. Most data repositories have an optional 12-month embargo period so researchers can share publicly at a later date once they’re happy with the overall project. The data repository acts as an additional backup to the host institutions’ own in case of data loss or calamity. By assuming the data and metadata can be sourced from a public location, the workflow developers ensure that the code they share won’t suffer from ‘file not found errors’ during reproduction. This ensures code and data are linked, which has been a priority for reproducibility [14] and a source of problems for shared code [7].

In addition to the experimental data, reference data that are critical to a project should be archived. For example, in the field of genomics, gene function annotation sets are relied upon for a common procedure called ‘pathway analysis’ [130]. These annotation sets are regularly updated, but versions are rarely reported [35] and finding historical data versions is sometimes difficult. In order to guarantee future reproducibility, snapshots of those reference data should be archived and shared if the license permits it.

### Documentation

Documentation is the glue that binds a data science project together. The published article is the central artifact that outlines the research project and links to the supporting materials. From a reproducibility standpoint, the methods section is the most critical part. It should be detailed enough so that other researchers can understand and replicate the experiments/analysis and yield similar results. Unfortunately, key details in bioinformatics data processing procedures are often omitted, which limits their reproducibility [22, 44, 131, 132]. Commonly missing information includes versions of software and packages as well as any parameter setting and configuration files. The ‘Materials Design Analysis Reporting’ (MDAR) checklist for authors has been developed to assist in comprehensive methodological reporting in the life sciences [133], and ‘Minimum Information About a Bioinformatics Investigation’ guidelines describe good reporting practices for computational biology research [13].

Researchers should consider depositing their thorough laboratory/*in silico* protocols as separate outputs to relevant repositories such as protocols.io, RIO Journal or Zenodo, minting a digital object identifier (DOI) that can be cited in the article. This is particularly helpful when there are strict word limits on articles.

The article should have clear links to supporting materials including datasets, software code and other resources like computational environments.

The code repository should have a detailed README file that plays a critical role in reproducibility. It should outline the purpose of the code/software/overall project and how it relates to the article. For example, some articles may rely on more than one repository for various parts of the research project, so these need to be explained.

The README should outline exactly what is required in order to reproduce the analysis, including the requirements and the instructions to reproduce. Typically, it is written in the Markdown format, which should be familiar to those using R Markdown or Jupyter notebooks. Hardware requirements such as RAM, CPU architecture and GPU needs need to be outlined. Software requirements, such as operating system, dependencies, container engine, workflow manager, etc., also need to be described. The exact instructions for reproduction should be clearly outlined. This should include what the output objects of the analysis are and instructions on how to access the results and what should be expected. Literate programming allows for thorough documentation of workflow methods and results that makes it more accessible for reproducers to comprehend the workflow details, which is not possible with standard code comments [134]. These instructions should be tested whenever changes are made to the codebase.

As outlined above, one of the goals is to reduce the complexity of reproduction, in particular by minimizing the number of commands required. This also makes the README documentation much simpler. For example, the pathway analysis workflow we previously developed could be reproduced and inspected with just six commands, starting from a new Ubuntu Linux installation [135]. By minimizing the complexity of reproduction and writing the instructions clearly in the README, reproducibility is made possible to a larger segment of the computational research community, especially those who are not experts in the languages used.

In addition, the README should outline the contents of the repository, how users can contribute to the project and how to report issues, such as code errors and discrepancies. The README should also list the contributors to the codebase and acknowledge any financial support. It may also include a link to the research article, citation instructions and a mention of the software license. A recently described set of recommendations for documenting software describes further measures that data analysts could use to enhance their code documentation [136].

The need for documentation extends to the other supporting information. The deposited data set needs thorough descriptive metadata to provide context. The container image and code snapshot should be deposited to a persistent repository with a thorough description of their purpose. Both the data and container image descriptions need to contain links to the DOI of the article, so that machines or individuals browsing these resources can understand the context of these objects relative to the overall research project.

### Continuous validation

When these principles are not integrated correctly and checked, problems can readily emerge [6, 7, 137]. This is why regular code testing after making updates to the code or data is considered best practice [22, 134, 138, 139]. For bioinformatics software, testing may involve a suite of unit tests of each function and another integration test whether the functions are working properly together [140].

For data analysts, the situation is somewhat different as a typical bioinformatics workflow might involve hundreds of lines of code, so conducting tests at each step in the process would be impractical. However incorporating such tests at key points in a workflow such as during quality control of input data, after data cleaning, before statistical analysis and a summary of the findings. While if/else statements can be used for testing, it is recommended to use a dedicated testing package such as testthat for R [141] or pytest for Python [142]. In the example in Box 1, testthat raises an error to halt the script if the test fails, while the if/else statement requires an extra line to do this.

**Box 1:** An example of a test in R using an if/else statement and with the testthat package. The test is checking that the iris dataset describes three species.

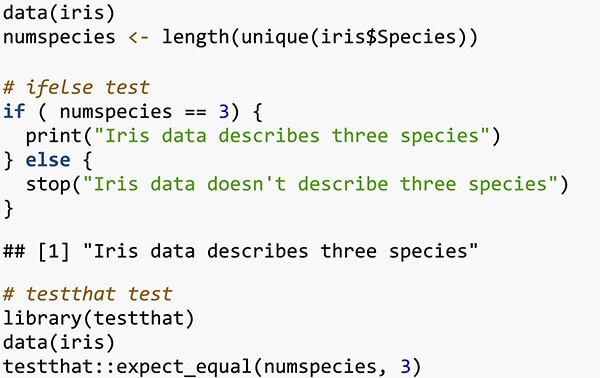

Literate programming also provides an opportunity to record sanity checks in an output report. In R, commands like dim(), length(), head(), str() are a simple way to show the features of a dataset to validate that data transformation steps are working as expected. Histograms, box plots, scatter plots and other simple charts are similarly effective.

Successful execution of all literate scripts in a project is an important validation step that proves that the scripts are free from code errors. It is worthwhile to conduct such testing on a separate computer to understand how others would fare reproducing such a workflow. Testing extends to documentation as well, so it is important to update it so it remains in accordance with the codebase, and actively seek feedback from others on its suitability.

In software development, continuous integration/continuous delivery (CI/CD) is a process of automated software compilation and testing. A derivation of this for data analysis has been termed ‘continuous analysis’ and involves automatic execution and testing whenever a change is made to a project’s code base [20, 143]. We prefer the term ‘continuous validation’ as it places more emphasis on the correctness of analyses to ensure they are fit to share. If a workflow is executed successfully and tests are passed, the repository and linked research article can be decorated with a ‘badge’ to indicate computational reproducibility, similar to badges used for other Open Science practices [144]. If those tests are designed cleverly such that certain conclusions drawn in the corresponding research article can be assessed computationally, then such tests could be used to demonstrate computational reproducibility without the need for individuals to execute the workflow themselves [145].

## CHALLENGES

Although the technological tools underlying these best practices have existed for a decade, progress on implementing them into computational research publications has been minimal. There are very many challenges, some of which have been discussed before [14, 18, 146, 147].

Due to the relative lack of systematic reproducibility/replication studies in bioinformatics, the severity of the reproducibility problem isn’t well appreciated. The studies that have been conducted point to reproducibility rates of ~20% in computational life sciences [5–7], but further studies are required to bring attention to the issue.

Journals are partly responsible, as their editorial policies influence author behaviors [148]. Except for a few outliers, verification of computational reproducibility is not in the scope of peer review, but automating this in the publication process should be considered. As others have noted, it is in the interests of funders to provide the infrastructure to facilitate this [14].

Another hindrance to reproducibility more broadly is the fixation on novel research directions rather than meticulous confirmation of previous findings [44, 146, 149–151]. This is a direct result of research assessment policies that incentivize journal-level prestige metrics and number of publications rather than rigor [146, 152]. A change in research assessment (‘e.g. track-record’) that recognizes and credits contributions toward reproducibility and rigor would help decrease funds wasted on sloppy research. Such changes would be welcomed by industry as they would face fewer replication failures [153], which could speed development of research into useful products.

Individual researchers also face difficulties. Life science graduates often receive inadequate training in data science that places them in a disadvantage considering the data-intensive nature of contemporary biology. This is compounded by the strong demand for data science experts in industry and government which leads to a deficit of academics fluent in data science [154]. This makes it challenging for organizations to recruit and retain capable data analysts and cultivate a community of practice. Addressing this problem is not straightforward. It requires enhancement of reproducible data science skills in undergraduate life science courses, investment in up-skilling the current workforce and offering working conditions that are competitive with other sectors. Specific workshop-based training for current researchers could help [155]. The Software Carpentry initiative is focused on this mission [156].

A lack of documented protocols and tutorial materials for highly reproducible analysis makes it difficult for researchers to confidently update their code development practices. Expert bioinformaticians can help by publishing comprehensive beginner-friendly step-by-step guides for frequently used routines. This approach is highly scalable, and the reach of open-access publishing ensures these materials are broadly accessible. For example, our group has just released such a protocol that walks novice users through the process of implementing functional enrichment analysis of gene expression data with the five pillars principles [135]. More guides like this are needed to make extreme reproducibility mainstream. A compendium of publicly available learning materials around the five pillar concepts are provided in Supplementary Information available online at http://bib.oxfordjournals.org/.

## CONCLUSION

As the chemotherapeutics case study highlights, there are significant risks to poorly conducted bioinformatics research, and current standards are falling short. If adopted widely, the transparency and reproducibility measures proposed here will reduce the chance of such disasters from happening again. Researchers, their institutions, publishers and funders each have a major role to play in fighting the reproducibility crisis by encouraging highly reproducible research practices.

**Key Points**

- Irreproducibility of bioinformatics studies remains a significant and still-relevant problem.
- We present the five pillars framework, a set of best practices that enable extremely reproducible workflows.
- Widespread adoption of these principles will enhance research reliability and will speed translation of basic research to tangible benefits.

## Supplementary Material

practical_guides_2023-08-31_bbad375

## Data Availability

Code repository: https://github.com/markziemann/5pillars.

## Author Biographies

**Mark Ziemann** is Head of Bioinformatics at Burnet Institute and former Lecturer in Biotechnology and Bioinformatics at Deakin University. His research spans bioinformatics as applied to omics data analysis in biomedicine, as well as aspects of research rigor.

**Pierre Poulain** is an Associate Professor at Université Paris Cité, Institut Jacques Monod, France. Research directions include bioinformatics, large-scale data analysis and education/pedagogy.

**Anusuiya Bora** is a PhD student at Deakin University School of Life and Environmental Sciences. Her research project involves improving the reproducibility of commonly used bioinformatics tools.
