# Supplementary material for: The five pillars of computational reproducibility: bioinformatics and beyond
Source: Brief Bioinform. 2023 Oct 21;24(6):bbad375. doi: 10.1093/bib/bbad375 (PMC10591307; doi:10.1093/bib/bbad375)
Supplement: practical_guides_2023-08-31_bbad375 [file practical_guides_2023-08-31_bbad375.docx]

Practical guides for implementing ‘Five Pillar’ principles

Mark Ziemann, Pierre Poulain, Anusuiya Bora

2023-08-31

The five pillar approach is a synthesis of over a decade of learning around computational reproducibility, and contains seven key recommendations and many best practices. These may be overwhelming for any data scientists, especially beginners and novices, so here we have tried to put together some recommended resources to enable practitioners to put these principles into practice. One of the questions practitioners may have is *“Where do I start?”* and *“In what order should I learn/master these principles?”*. The following sections have been arranged in order, and have been submitted to the [Internet Archive](https://archive.org/) for posterity.

## Getting started with data analysis in R and Python

Learn the basics of your scripting language (R/Python/shell) so that you can do useful analyses with it. Learn by solving problems.

### Introduction to R and Rstudio

- [Video: Rstudio for the Total Beginner](https://www.youtube.com/watch?v=FIrsOBy5k58)
- [Video: Running Basic Statistical Analysis in R](https://www.youtube.com/watch?v=I6FJo8x1wZE)
- [Ebook: R for data science](https://r4ds.had.co.nz/)
- [Ebook: YaRrr! The Pirate’s Guide to R](https://bookdown.org/ndphillips/YaRrr/)

### Introduction to Unix and shell programming

- [Ebook: Bioinformatics Data Skills](https://vincebuffalo.com/book/)
- [Book: Unix Shell Programming (ISBN:0672324903)](https://www.amazon.com/Unix-Shell-Programming-Stephen-Kochan/dp/0672324903)

### Introduction to Python

- [Tutorial: Beginner’s Guide to Python](https://wiki.python.org/moin/BeginnersGuide)
- [Ebook: The Absolute Beginner’s Guide to Python Programming](https://doi.org/10.1007/978-1-4842-8716-3)
- [Video: Python Tutorial for Absolute Beginners](https://www.youtube.com/watch?v=Z1Yd7upQsXY&t=19s)

### Introduction to VS Code

- [Tutorial and video: Getting Started with Visual Studio Code](https://code.visualstudio.com/docs/introvideos/basics)

### Introduction to Galaxy: bioinformatics in the browser

- [Galaxy: A very short introduction](https://www.youtube.com/watch?v=VZoz3k5EehI&ab_channel=GalaxyProject)

## Literate programming with R Markdown, Jupyter and Quarto

Convert those scripts into literate scripts and document them with introduction, methods, code comments, results observations and interpretations. Use JupyterLab (Jupyter notebook) or Rstudio (Rmarkdown) development environments to provide graphical interfaces to the programming languages.

- [Tutorial: Beginner Guide to Markdown](https://medium.com/@itsjzt/beginner-guide-to-markdown-229adce30074)
- [Tutorial: Getting Started with R Markdown — Guide and Cheatsheet](https://www.dataquest.io/blog/r-markdown-guide-cheatsheet/)
- [Ebook Chapter: Reproducible Workflow with R Markdown](https://tysonbarrett.com/Rstats/chapter-9-reproducible-workflow-with-rmarkdown.html)
- [Tutorial: Reproducibility and R Markdown](https://andrewbtran.github.io/NICAR/2017/reproducibility-rmarkdown/rmarkdown.html)
- [Quick guide: R based literate scripts with Quarto](https://quarto.org/docs/computations/r.html)
- [Quick guide: Python based literate scripts with Quarto](https://quarto.org/docs/computations/python.html)
- [Tutorial: Jupyter/IPython Notebook Quick Start Guide](https://jupyter-notebook-beginner-guide.readthedocs.io/en/latest/)
- [Tutorial: How to Use Jupyter Notebook](https://www.dataquest.io/blog/jupyter-notebook-tutorial/)
- [Tutorial: The Ultimate Beginner’s Guide to Jupyter Notebooks](https://medium.com/velotio-perspectives/the-ultimate-beginners-guide-to-jupyter-notebooks-6b00846ed2af)
- [Tutorial: Write your next paper in MyST Markdown with data, code & Jupyter notebooks](https://www.youtube.com/watch?v=1BWYLzQd8Ds&t=4s&ab_channel=curvenote)

## Practical guides for git and GitHub

Learn how integrate version control into your development environment.

- [Tutorial: GitHub and Git for Beginners](https://www.datacamp.com/tutorial/github-and-git-tutorial-for-beginners/)
- [Tutorial: How to Use Git and GitHub – Introduction for Beginners](https://www.freecodecamp.org/news/introduction-to-git-and-github/)
- [Video Tutorial: Git and GitHub for Beginners - Crash Course](https://www.youtube.com/watch?v=RGOj5yH7evk)
- [Video Tutorial: Git basics - 4 short video tutorials](https://git-scm.com/videos)
- [Quick Guide: Git for humans](https://speakerdeck.com/alicebartlett/git-for-humans)
- [Tutorial: How to use Git with R and RStudio](https://www.geo.uzh.ch/microsite/reproducible_research/post/rr-rstudio-git/)
- [Tutorial: How to link and use Git with RStudio](https://anderfernandez.com/en/blog/how-to-link-and-use-git-with-rstudio/)
- [Ebook: Happy Git and GitHub for the useR](https://happygitwithr.com/index.html)
- [Ebook: Reproducible workflow and version control with Git and Github](https://jules32.github.io/2016-07-12-Oxford/git/)
- [Tutorial: Introduction to Using Git in Jupyter](https://andgarc.github.io/basic-git-jupyter-lesson/index.html)
- [Tutorial: How to use Git / GitHub with Jupyter Notebook](https://blog.reviewnb.com/github-jupyter-notebook/)
- [Ebook: Version Control with Git](https://swcarpentry.github.io/git-novice/)
- [Tutorial: Using Git source control in VS Code](https://code.visualstudio.com/docs/sourcecontrol/overview)

## Practical guides for Conda, Guix and Docker

Control the environment. Record the version of programming languages and packages. Become familiar and experiment with Conda, Guix and Docker. Select the option that works best for your project.

- [Tutorial: Getting started with conda](https://conda.io/projects/conda/en/latest/user-guide/getting-started.html)
- [Video tutorial: Anaconda Beginners Guide for Linux and Windows](https://www.youtube.com/watch?v=MUZtVEDKXsk)
- [Tutorial: Running RStudio Server with Docker](https://davetang.org/muse/2021/04/24/running-rstudio-server-with-docker/)
- [Tutorial: Creating a Docker Image for your Application](https://www.stereolabs.com/docs/docker/creating-your-image/)
- [Documentation: Getting started with GNU Guix](https://guix.gnu.org/manual/en/html_node/Getting-Started.html)

## Practical guides to data sharing

Learn about the FAIR principles and make the research data available in a public repository.

- [Guide: Dryad good data practices](https://datadryad.org/stash/best_practices)
- [Guide: Best practice for managing your outputs on Figshare](https://help.figshare.com/article/best-practice-for-managing-your-outputs-on-figshare)
- [Guide: Introduction to data sharing](https://doi.org/10.5281/zenodo.3518692)
- [Guide: FAIR Data (according to Australian Research Data Commons)](https://ardc.edu.au/resource/fair-data/)
- [Documentation: FAIRsharing.org](https://fairsharing.org/)

## Practical guides for documenting computational research

Document the process of reproduction as if you were an external researcher. Test the instructions.

- [Guide: How to write an Awesome README](https://towardsdatascience.com/how-to-write-an-awesome-readme-68bf4be91f8b)
- [Ebook: Bioinformatics Data Skills Chapter 2](https://vincebuffalo.com/book/)
- [Example: README for “A Reproducible Data Analysis Workflow with R Markdown, Git, Make, and Docker”](https://github.com/aaronpeikert/reproducible-research/blob/master/README.md)
- [Guide: A Quick Guide to Organizing Computational Biology Projects](https://journals.plos.org/ploscompbiol/article?id=10.1371/journal.pcbi.1000424)

## Extend the scripts to make them end-to-end processes

Try to extend the scripts to make them end-to-end processes, by linking to raw data and outputting whole figures or research articles.

- [Ebook: The Practice of Reproducible Research - Case Studies and Lessons from the Data-Intensive Sciences](http://www.practicereproducibleresearch.org/)
- [Ebook: The Turing Way - Guide for Reproducible Research](https://the-turing-way.netlify.app/reproducible-research/reproducible-research.html)
- [Ebook: Building reproducible analytical pipelines with R](https://raps-with-r.dev/)
- [Documentation: The {targets} R package user manual (workflow manager for R)](https://books.ropensci.org/targets/)
- [Video Tutorial: Reproducible Data Analysis with Snakemake](https://www.youtube.com/watch?v=hPrXcUUp70Y)
- [Tutorial: Understanding Snakemake](https://vincebuffalo.com/blog/2020/03/04/understanding-snakemake.html)
- [Tutorial: An example Snakemake workflow](https://snakemake.readthedocs.io/en/stable/tutorial/basics.html)
- [Quick Guide: Using snakemake to do simple wildcard operations on many, many, many files](http://ivory.idyll.org/blog/2021-snakemake-simple-operations.html)
- [Tutorial compendium: Learn Nextflow in 2023](https://snakemake.readthedocs.io/en/stable/tutorial/basics.html)

## Continuous analysis/continuous validation

Continuous analysis involves automatic execution and testing of code, which is important when changes are made to code or data. Results generated in HTML are automatically updated. Badges on the repository indicate the status of the codebase in terms of completing with or without errors. Transparency and reproducibility help validation which can be continuously maintained.

- [Ebook: Building reproducible analytical pipelines with R](https://raps-with-r.dev/)
- [Ebook: The Turing Way - Guide for Reproducible Research](https://the-turing-way.netlify.app/reproducible-research/reproducible-research.html)
- [Tutorial: Continuous Analysis](https://greenelab.github.io/continuous_analysis/)
- [Tool: Drone: System for continuous delivery](https://www.drone.io/)
- [Documentation: GitHub Actions](https://docs.github.com/en/actions)
